# COVID-19 und die Geschichte der sozialwissenschaftlichen Katastrophenforschung

**DOI:** 10.1007/s00048-020-00254-8

**Published:** 2020-05-07

**Authors:** Cécile Stephanie Stehrenberger

**Affiliations:** Institut für Geschichtswissenschaft, TU Braunschweig, Schleinitzstr. 13, 38106 Braunschweig, Deutschland

**Keywords:** COVID-19, Katastrophenforschung, Sozialwissenschaften, Katastrophen, COVID-19, disaster research, social sciences, disaster

## Abstract

Dieser Beitrag ist Teil des Forums COVID-19: Perspektiven in den Geistes- und Sozialwissenschaften. Mit Blick auf die sozialwissenschaftliche Katastrophenforschung des Kalten Krieges als Herkunftsort und als historisches Vergleichsmoment beschäftigt sich der Artikel mit der bisherigen sozialwissenschaftlichen Auseinandersetzung mit der COVID-19-Krise. Er behandelt erstens, wie die Rolle von sozialer Ungleichheit erörtert wird, zweitens die Idee der Katastrophe als „große Enthüllerin“ und drittens das Verhältnis von Katastrophenwissenschaft, Öffentlichkeit und Politik.

## Abstract (en)

This paper is part of Forum COVID-19: Perspectives in the Humanities and Social Sciences.

The article connects and juxtaposes current day (social scientific) discussions on the social effects of the COVID-crisis with the history of social science disaster research during the Cold War. It elaborates on statements regarding the role of social inequalities in disaster, the idea of the “revealing” nature of disaster and crisis, and the relationship between disaster science, public and politics.

[…] die historische Zäsur der Covid-19-Pandemie verändert das Leben und Arbeiten der Menschen gerade in grundstürzender Weise. Von den erdrutschartigen Ereignissen zunächst überrumpelt, sind die Sozialwissenschaften nunmehr dabei, sich neu zu organisieren. Denn trotz des aktuell notwendigen Fokus auf medizinische Fragen ist es geboten, auch die gesellschaftlichen Implikationen von Corona mit systematischer Forschung zu begleiten,

schrieb am 26. März 2020 der Journalist Christoph David Piorkowski im *Tagesspiegel* und berichtete von drei verschiedenen sozialwissenschaftlichen Erhebungen, die Berliner Einrichtungen aktuell durchführen oder planen (Piorkowski 2020).

Die Diagnose, die Covid-19-Pandemie bedeute eine „historische Zäsur“ und wäre ein „überrumpelndes“ „Ereignis“ ohne Präzedenz, tauchte im März und April 2020 vielerorts auf. Sie ist, gerade was die Produktion sozialwissenschaftlichen Wissens betrifft, nur bedingt richtig.

Schon in den frühen 1950er Jahren begann von den USA aus eine Reihe von teilweise Armee-finanzierten *Social Science Disaster Research*-Gruppen hunderte von Feldstudien nach Natur- und Technikkatastrophen durchzuführen, um die Reaktionen von Individuen, Organisationen und *communities* auf sie „empirisch“ zu untersuchen. Ziel war es, praktisches Wissen zu generieren, das sich in Katastrophen und Krisensituationen (einschließlich Kriegen) verschiedenster Art einsetzen ließ. Schnell kamen ihre Mitglieder – größtenteils Anthropolog*innen und Soziolog*innen – zu dem Schluss, Menschen würden sich in Katastrophen mehrheitlich rational und solidarisch verhalten und an ihnen sogar wachsen. Als Vordenker*innen der „Resilienz“-Idee, aber auch anderweitig haben diese Katastrophenforscher*innen in den vergangenen Jahrzehnten entscheidend Vorstellungen davon mitgeprägt, wie sich Menschen in Katastrophen und Krisen (zu) verhalten (haben) (Stehrenberger 2017; Stehrenberger 2016). Vorstellungen, die auch im bisherigen Umgang mit der Corona-Krise zum Einsatz gekommen sind. Indem ich auf die *social science disaster research* des Kalten Krieges als Herkunftsort aber auch als historisches Vergleichsmoment blicke, setzte ich mich im Folgenden erstens damit auseinander, wie bisher die Rolle von sozialer Ungleichheit in COVID-19 (sozialwissenschaftlich) erörtert worden ist. Zweitens behandle ich die Idee der Katastrophe als „große Enthüllerin“, bevor ich mich drittens dem Verhältnis von Katastrophenwissenschaft, Öffentlichkeit und Politik zuwende.

## Vor dem Virus sind (nicht) alle gleich

Zu Beginn der Verbreitung des Corona-Virus in Europa standen vor allem „Risikogruppen“, bei deren Angehörigen eine Infektion besonders schwer verlauf würde, im Fokus der öffentlichen Aufmerksamkeit. Bald schon stellten aber Politiker*innen klar, dass der Virus nicht zwischen Menschen unterscheide, sondern jede/r es kriegen könne. Anfang März sprach auch unter anderem der Ökonom Branko Milanovic vom Virus als „Gleichmacher“, der nun auch Menschen aus reichen Ländern mit Freiheitsbeschränkungen belasten würde, die für Bewohner*innen des Irans und Chinas längst Normalität wären (Milanovic 2020). Die Rede vom „great equalizer“ verlor allerdings schon an Popularität, bevor US-amerikanische Großstädte besonders viele *African* und *Hispanic Americans* unter ihren Corona-Toten verzeichneten. In Deutschland waren es in wissenschaftlichen Kontexten vor allem Geschlechterforscher*innen, die speziell mit Blick auf *care work,* betonten die Pandemie wäre für bestimmte Menschengruppen nicht nur medizinisch, sondern auch sozial besonders schädlich und verstärke bereits bestehende soziale Ungleichheiten – auch solche unter Frauen (Carstensen et al. 2020).

Der Diskussionsverlauf in der COVID-19 Krise weist hier Parallelen aber auch markante Unterschiede zu jenem auf, der sich von Anfang der 1950er bis Mitte der 1980er Jahre in der sozialwissenschaftlichen Katastrophenforschung vollzog. Auch hier war zu Beginn die Rede davon, dass Krisen und Katastrophen soziale Ungleichheiten kurzfristig nivellieren würden. Ab den 1960er Jahren beobachteten Forscher*innen dann vermehrt, dass strukturelle Ungleichheiten zwischen weißen Amerikaner*innen und Minderheiten sich negativ darauf auswirkten, wie eine Bevölkerung insgesamt mit einer Katastrophe zurechtkam, und ausschlaggebend dafür waren, ob ein Katastrophen-Agent (etwa eine Flut) für eine Bevölkerung(sgruppe) überhaupt zu einer Katastrophe wurde. Ab den 1970er Jahren benutzten die *disaster researcher* immer häufiger den Terminus der sozialen „Vulnerabilität“, um diese Sachverhalte zu beschreiben. Zu einem regelrechten „Paradigma“ wurde dieser Begriff in den 1980er Jahren im Umgang mit „Entwicklungsländern“. Deren Bewohner*innen erhielten einen pauschalen Opfer-Status, der die Annahme affirmierte, westliche/nördliche Akteure müssten mit Blick auch schon auf zukünftige Katastrophen dort „helfend“ intervenieren (Bankoff et al. 2004).

## Die große Enthüllung

Für die COVID-19-Pandemie ist in den vergangenen Wochen auch oft zu lesen gewesen, dass sie nicht nur von Ungleichheiten geprägt wäre, sondern solche in ihr besonders gut sichtbar würden – die Katastrophe als Brennglas oder als Kontrastmittel, das normalerweise verborgene Aspekte des Sozialen zu Tage fördert. Die *social science disaster research* hatte solchen Zuschreibungen spätestens ab den 1960er Jahren wissenschaftliche Autorität verliehen. So erklärte der Soziologe Charles Fritz in einem bis heute viel zitierten Artikel, in Katastrophen würden „vital social processes“ deswegen besonders sichtbar werden, weil sie in ihnen sehr schnell verliefen und sich von dem privaten in den öffentlichen Raum verlagerten. Er argumentierte weiter, Katastrophen seien „realistische Laboratorien“, welche die „integration, stamina, and recuperative power“ von Gesellschaften zur Anschauung brächten, indem sie diese „testen“ würden (Fritz 1961: 111).

Auch das sozialwissenschaftlich informierte Sprechen über den enthüllenden Effekt der COVID-19-Krise ist damit einhergegangen, dass diese als „test-case“, oder als ein „die Welt in ein riesiges Laboratorium“-transformierendes Phänomen bezeichnet wurde (Bhatt 2020). In einer aus der jüngeren Verwendung des Laborbegriffes in anderen Kontexten bekannten Weise (Guggenheim 2012: 11–12) sollte damit auch unterstrichen werden, dass die Katastrophe eine Chance bieten würde, neue Formen der Kooperation zu erproben.

Während der Vorschlag der französischen Mediziner*innen Jean-Paul Mira und Camille Locht Teststudien „in Afrika“ durchzuführen, entschieden mit dem Hinweis „Africa is not a testing lab“ zurückgewiesen wurde (Oteng 2020), ist die Idee der Katastrophe als sozial(wissenschaftlich)es Laboratorium bislang kaum auf Kritik gestoßen. Und dies obgleich, oder vielleicht gerade weil der Laborbegriff in seinem engeren Sinn an ein Ideal von (epistemischer) Kontrolle geknüpft ist (Guggenheim 2012: 3–5) und sich die COVID-19 Krise durch einen Verlust solcher Kontrolle ausgezeichnet hat (MacGregor 2020).

Zur Idee der Katastrophe als Enthüllerin – ob mit oder ohne Labor-Begriff – ist für COVID-19 anzumerken, dass diese Krise in Umkehr von Fritzens Herleitung zu einem Rückzug des Sozialen in den privaten Raum geführt hat, wo sie dem Blick bzw. der Datenerhebung der Sozialwissenschaftler*innen entzogen bleibt. Daran ändert auch die Erwerbsarbeit von zuhause aus im Zoom-Modus wenig. Gleichzeitig hat etwa die Desaster-STS-Forscherin Kim Fortun zu bedenken gegeben, die Aufmerksamkeitskonzentration auf die Pandemie laufe Gefahr andere Katastrophen zu überblenden (Knowles 2020b).

Mit Blick auf die Geschichte der sozialwissenschaftlichen *disaster research* lässt sich weiter einwenden, dass die dortige Postulierung des Enthüllungsgedankens – etwa in dem zitierten Artikel von Fritz – mit einer Definition von Katastrophen als disruptiven, in Raum und Zeit beschränkten Ereignissen einherging. In Abgrenzung von einem derartigen Katastrophenverständnis haben sich in der kulturwissenschaftlichen Analyse in den 1990er Jahren Konzepte wie das der „chronischen“ oder „schleichenden“ Katastrophe zu etablieren begonnen (Anderson et al. 2019). Sie erfassen Prozesse und Strukturen, die wie „slow violence“ (Nixon 2011), ihre destruktive Wirkung nur langsam entfalten und dabei zumeist unsichtbar bleiben.

Auch im Hinblick auf die COVID-19-Pandemie sind in den letzten Wochen vor allem in der Desaster-STS-Community oft Begriffe wie „slow disaster“ gefallen. Dabei wurde erklärt, ihre zerstörerische Kraft resultiere daraus, dass das katastrophale Ereignis der Pandemie auf die schleichenden Katastrophen Armut und Rassismus träfe. – Phänomene, die im Normalzustand gar nicht als Katastrophen erkennbar wären (Knowles 2020a).

Die tatsächliche Passfähigkeit dieses Umdenkens von Enthüllung und schleichender Katastrophe in Bezug auf die COVID-19-Krise wird sich noch erweisen. Anzumerken bleibt mit Thom Davies, dass schleichende Katastrophen auch ohne desaströse Ereignisse gerade für die von ihnen betroffenen Menschen in der Regel alles anderes als unsichtbar sind und es nicht an Stimmen mangelt, die über sie berichten (Davies 2019: 3). Nur bleiben diese Stimmen oft ungehört.

## Katastrophenwissenschaft, Politik und mediale Öffentlichkeit

Auch Hannah Holleman (2018) und Scott Knowles (2011) konstatieren für den historischen und gegenwärtigen Umgang mit Katastrophen ein häufiges und bewusstes Ignorieren gerade von wissenschaftlicher Erkenntnis und zwar insbesondere seitens politischer Entscheidungsträger*innen. Dass er dies für problematisch hält, dokumentiert der Historiker Knowles auch in seinen aktuellen COVID-19-Podcasts (Knowles 2020b).

In seinem „Coronavirus-Update“ vom 30. März 2020 drückte dagegen der Virologe Christian Drosten sein Missfallen über die mediale „Überzeichnung“ der Rolle der Wissenschaft in der Krise aus. Ihr falle es lediglich zu, Daten zu generieren und zu erklären: „Kein Wissenschaftler will überhaupt so Dinge sagen wie ‚diese politische Entscheidung, die war richtig‘ oder ‚diese politische Entscheidung, die war falsch‘ oder ‚diese politische Entscheidung, die muss jetzt als nächstes getroffen werden‘. Sie hören das von keinem seriösen Wissenschaftler“ (Martini 2020).

Drostens Äußerungen erinnern an das Unbehagen, welches anfänglich viele *disaster researcher* mit ihrer Funktion als Berater*innen an den Tag legten, die ihnen mit den Idealen unabhängiger, werturteilsfreier Wissenschaft schwer vereinbar schien. Sie bemühten sich, aus einem distanzierte Objektivität versprechenden Hintergrund zu beobachten, und begannen erst in der zweiten Hälfte der 1960er Jahre das öffentliche und direkte Liefern von Handlungsempfehlungen stärker in ihr wissenschaftliches Selbstverständnis zu integrieren.

Knowles und Drosten sprechen aber auch insofern aus verschiedenen Positionen heraus, als dass ihren Disziplinen bisher sehr unterschiedliche Autorität in der Erklärung der COVID-19-Krise zugestanden wurde. Bei den Rufen, es sollten jetzt Wissenschaftler*innen Entscheidungen über den Umgang mit der Krise treffen, (wogegen Drosten sich verwehrt hat), waren meist nicht die Sozial- oder Kulturwissenschaften gemeint. Die Gründe dafür liegen unter anderem just in der Wahrnehmung der Krise als ein nur in ihren Auswirkungen soziales und von der Normalität klar trennbares Phänomen.

Es gibt Anzeichen dafür, dass die Corona-Krise zum Anlass wird, diese Dynamik zwischen Katastrophenwissenschaftsordnungen und der Konzeptualisierung ihrer Gegenstände eingehender zu reflektieren (MacGregor 2020; Knowles 2020b). Zentral erscheint mir, dass sich in dieser Reflexion in Zukunft Lebens- und Sozialwissenschaftler*innen gemeinsam damit auseinandersetzen, welche Rolle die für den Verlauf der Corona-Krise so zentralen globalen und sozialen Ungleichheitsbeziehungen auch bei der Frage spielen, wem welche Art von Forum geboten wird.
